# Algorithmic fairness and bias mitigation for clinical machine learning with deep reinforcement learning

**DOI:** 10.1038/s42256-023-00697-3

**Published:** 2023-07-31

**Authors:** Jenny Yang, Andrew A. S. Soltan, David W. Eyre, David A. Clifton

**Affiliations:** 1 Institute of Biomedical Engineering, Department of Engineering Science, University of Oxford, Oxford, UK; 2 John Radcliffe Hospital, Oxford University Hospitals NHS Foundation Trust, Oxford, UK; 3 RDM Division of Cardiovascular Medicine, University of Oxford, Oxford, UK; 4 Big Data Institute, Nuffield Department of Population Health, University of Oxford, Oxford, UK; 5 Oxford-Suzhou Centre for Advanced Research, Suzhou, China

**Keywords:** Medical ethics, Translational research, Diagnosis

## Abstract

As models based on machine learning continue to be developed for healthcare applications, greater effort is needed to ensure that these technologies do not reflect or exacerbate any unwanted or discriminatory biases that may be present in the data. Here we introduce a reinforcement learning framework capable of mitigating biases that may have been acquired during data collection. In particular, we evaluated our model for the task of rapidly predicting COVID-19 for patients presenting to hospital emergency departments and aimed to mitigate any site (hospital)-specific and ethnicity-based biases present in the data. Using a specialized reward function and training procedure, we show that our method achieves clinically effective screening performances, while significantly improving outcome fairness compared with current benchmarks and state-of-the-art machine learning methods. We performed external validation across three independent hospitals, and additionally tested our method on a patient intensive care unit discharge status task, demonstrating model generalizability.

## Abstract (web summary)

The tendency of machine learning algorithms to learn biases from training data calls for methods to mitigate unfairness before deployment to healthcare and other applications. Yang et al. propose a reinforcement-learning-based method for algorithmic bias mitigation and demonstrate it on COVID-19 screening and patient discharge prediction tasks.

## Main

Advancements in computational resources and the availability of vast amounts of digital health data are revolutionizing our understanding of general and personalized health assessment. While machine learning (ML)-based technologies offer clear benefits, it is crucial to ensure the fairness and equity of models, particularly in healthcare settings where algorithmic findings directly influence clinical decision-making and patient care. Ideally, a model should extract useful generalizations from the data without exhibiting any form of unfair discrimination. By achieving this, the model’s performance improves, while fostering trust among clinicians and patients in its effectiveness and reliability.

ML models are prone to bias based on the composition of training data, leading to unfair differences in performance for specific subgroups in predictive tasks. These biases hinder a model’s ability to accurately capture the relationship between features and the target outcome, resulting in poor generalization across subgroups and unfair decision-making^1–5^.

Prior works on ML fairness have established statistical fairness metrics such as statistical parity, equalized odds, equal opportunity and test fairness to evaluate notions of fairness^2,3,5–7^. Thus, fairness-aware ML methods aim to improve on such fairness definitions.

In this study, our focus is on optimizing for equalized odds. Consider a binary classifier that predicts labels *y*_*i*_ ∈ {0, 1} for samples *i* with features *x*_*i*_. A subgroup of samples *Z* is considered sensitive (that is, a group that a model may be biased against) compared with a non-sensitive complement *Z*′, whereby *Z*/*Z*′ represents subgroups from a real-world attribute, such as ethnicity, socioeconomic community or gender. Following the definition of equalized odds, a classifier $\hat{Y}$ is fair if $\hat{Y}$ and *Z* are conditionally independent given *Y* (refs. ^2,3,5,6^). For binary classification, this is equivalent to $P(\hat{Y}=1| Y=y,Z=0)=P(\hat{Y}=1| Y=y,Z=1),y\in \{0,1\}$. This definition can also be extended to multi-class classification.

In the context of fairness and bias, our focus is on clinical applications for three key reasons. Firstly, a biased model can lead to inaccurate predictions for crucial and potentially life-altering decisions. Secondly, bias against certain groups can result in disparities in the quality of care received by patients from those groups compared with others. Lastly, a biased model has the potential to worsen existing healthcare and societal inequities^3^. These factors collectively undermine clinician and patient trust, making the deployment of ML models in clinical practice more challenging.

Ethnicity-related unfair bias is a prominent concern in healthcare. It can inadvertently occur due to admission bias, volunteer bias, sampling bias or observer bias during data collection, resulting in data that do not represent the general population^8,9^. ML models have previously shown susceptibility to ethnicity-based biases. For instance, a study revealed a recidivism prediction model that exhibited bias against black defendants, wrongly classifying them as future criminals at nearly double the rate of white defendants^10^. In clinical applications, researchers have found unequal performance of ML models across different patient populations^11^, which can lead to negative consequences for under-represented groups^12^. This issue is particularly pertinent as sample populations used in studies may not adequately represent the overall patient population because of limited resources, regional biases and other factors. For example, randomized trials evaluate treatment effects for a trial population; however, participants in clinical trials are often demographically unrepresentative of the patient population that ultimately receives the treatment^11,13^. Consequently, if a model determines who receives a specific drug or intervention, minority groups (for example, ethnic minorities, women and obese patients) might receive the least, perpetuating demographic inequities in healthcare. Privacy preservation and statistical disclosure are also affected because regions with a small number of patients from a particular ethnicity face increased identification risk if the ML model exhibits bias against that group^3^.

Inequities can also arise between different healthcare centres, as they can exhibit variations in disease prevalence, mortality rates, quality of healthcare services, and the use of specific medical devices^14–18^. ML models trained on real-world data from one hospital may not generalize to new settings due to unintentional site-specific biases introduced during data collection, processing and organization (known as measurement bias)^19^. For instance, a study found that state-of-the-art computer vision methods consistently underdiagnosed underserved patient populations^1^. To mitigate such biases, many ML-based clinical projects aim to combine datasets from multiple hospitals to increase the training data volume, as generalizability often requires large datasets. However, models can still acquire site-specific biases during training due to varying amounts of available training data across different centres. If these biases influence a model’s decisions, certain hospitals may experience inferior outcomes, widening inter-hospital inequities and discouraging the adoption of ML-based technologies^3^.

Because of these concerns, there is growing attention being given to ML fairness and bias mitigation, with practitioners typically employing techniques at either the data, algorithm or evaluation level. For the purposes of our study, we specifically focus on an algorithmic-level technique, whereby we aim to develop a fair model using a reinforcement learning (RL) paradigm. The current literature for addressing bias mitigation at the algorithmic level has primarily focused on standard supervised learning using adversarial debiasing—a technique where a model is trained to learn parameters that do not infer sensitive features. Here, a predictor network is trained against an adversary network, where the adversary assures that the predictor’s output is not correlated with the specified sensitive feature (that is, the unwanted bias that we are trying to mitigate). A fairness metric can also be imposed as a constraint or incorporated into a loss function^6^ (Supplementary Section B). This technique has been used to develop models that output fair predictions and has previously been successful in reducing gender (male versus female) bias in salary prediction^6,20^ and ethnicity (black versus white) bias in recidivism prediction^21^. Adversarial models have also been used to effectively predict COVID-19, whilst simultaneously improving outcome fairness with respect to site (hospital)-specific and ethnic biases^3^. As specialized methods have been shown to be necessary for mitigating unwanted biases, we aimed to use an RL framework (instead of an adversarial one) to optimize fairness outcomes.

**Fig. 1:**
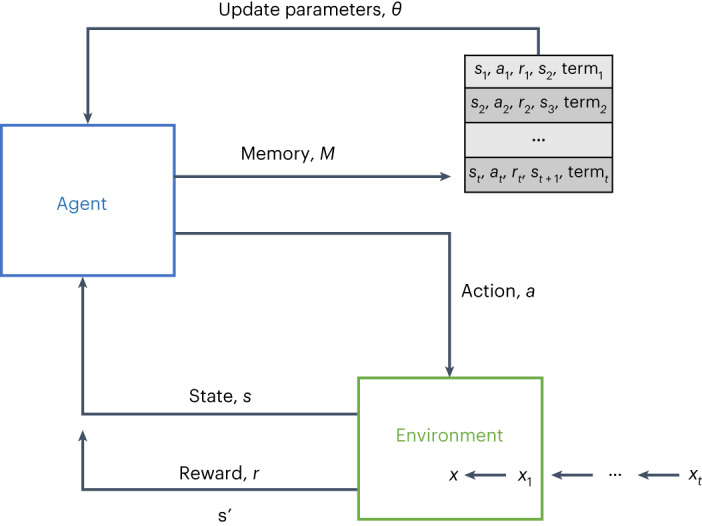
Reinforcement learning framework. Overview of the general RL framework used. For this study, the ‘agent’ used is a duelling *Q*-network, and the ‘environment’ is the features of each sample.

RL—whereby an agent interacts with an environment to learn a task—has been linked to many real-world artificial intelligence successes, with many well-known exemplars in gameplay and control. However, the core elements of RL have been shown to be successful on a wider range of tasks, including those which, on the surface, do not appear to have a particular ‘agent’ interacting with an ‘environment’ (which is typically regarded as the standard RL set-up^22,23^). Such problems include classification tasks, which have commonly been addressed using standard supervised learning algorithms (where an input is mapped through a model to predict a class label). RL, instead, uses an agent to interact with the input to determine which class it belongs to and then receives an immediate reward from its environment based on that prediction. A positive reward is given to the agent when a label is correctly predicted and a negative one is given otherwise. This feedback helps the agent learn the optimal ‘behaviour’ for classifying samples correctly, such that it accumulates the maximum rewards. To do this, an agent performs actions that set memory cells, which can then be used by the agent (together with the original input) to select actions and classify samples^24^. Specialized reward functions have previously been successful in mitigating large data imbalances with respect to the predicted label^25,26^. Thus, instead of focusing on label imbalance, we aimed to formulate a deep RL framework with the specific purpose of improving algorithmic fairness and mitigating unwanted biases (Fig. 1).

**Fig. 2:**
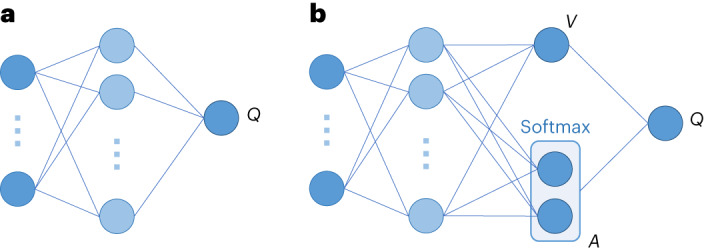
Single-stream and duelling *Q*-network comparison. **a**, A typical single-stream *Q*-network. **b**, A duelling architecture, with two streams to independently estimate the state values (scalar) and advantages (vector) for each action (this implements equation (7)).

Specifically, we developed a duelling double-deep *Q*-network (DDQN; Fig. 2) and evaluated our method on a real-world, clinical task—COVID-19 screening using anonymized electronic health record data from hospital emergency rooms. For this task, we aimed to mitigate any unwanted ethnicity-based and site (hospital)-specific biases. To demonstrate the utility of our method across diverse clinical tasks, we performed additional analyses on a patient discharge status task using electronic health record data from intensive care units (ICUs). Although we use clinical case studies, the framework introduced can be generalized across many different domains and can be applied to a variety of tasks and features.

## Results

In this study, we introduced a method for training fair, unbiased ML models, based on a deep RL framework. We evaluated it on two complex, real-world tasks—screening for COVID-19 and predicting patient discharge status—while aiming to mitigate site (hospital)-specific and demographic (patient ethnicity) biases. Through comparison of our RL method against current benchmarks and state-of-the-art ML methods—RL (without a debiasing component), XGBoost and a fully connected neural network (NN; both with and without cost-adjusted weights inversely proportional to the frequency of sensitive attributes) and adversarial debiasing—we found that RL demonstrably improved outcome fairness, while still achieving strong classification performance.

### Debiasing ethnicity

After training models on patient cohorts from Oxford University Hospitals National Health Service (NHS) Foundation Trust (OUH), we externally validated our models across three external patient cohorts from Portsmouth Hospitals University NHS Trust (PUH), University Hospitals Birmingham NHS Trust (UHB) and Bedfordshire Hospitals NHS Foundation Trust (BH). All models achieved reasonably high area under receiver operator characteristic curve (AUROC) scores across all test sets (Table 1), comparable to those reported in previous studies^3,19,26,27^ (which used similar patient cohorts and features; Supplementary Table 13), demonstrating that we trained strong classifiers to begin with. AUROC scores for predicting COVID-19 status stayed relatively consistent across all test sets, achieving the highest performances on the BH cohort (PUH: AUROC range 0.834–0.882 (confidence interval, CI: 0.821–0.894); UHB: 0.834–0.868 (0.807–0.892); BH: 0.897–0.923 (0.861–0.954)). With respect to the model used, all models achieved similar AUROCs; however, the highest AUROCs were generally achieved by the standard supervised learning models—adversarial and both weighted and unweighted XGBoost and NN models (mean AUROCs of 0.869 (RL), 0.857 (RL, unweighted), 0.881 (adversarial), 0.885 (NN), 0.881 (XGBoost), 0.887 (NN, weighted), 0.879 (XGBoost, weighted)). Using a sensitivity configuration of 0.9, we obtained consistent scores for sensitivity across all models and cohorts (PUH: sensitivity range 0.872–0.919 (CI: 0.855–0.933); UHB: 0.862–0.879 (0.825–0.913); BH: 0.862–0.935 (0.805–0.976)), with RL achieving the highest sensitivities on the UHB and BH test sets (however, it should be noted that RL had either the lowest or second-lowest specificities). And, as seen in previous studies, our models achieved high prevalence-dependent negative predictive value (NPV) scores (>0.978), demonstrating the ability to exclude COVID-19 with high confidence. Furthermore, these results demonstrate that an RL paradigm is more generalizable in diverse environments, with superior AUROC for the BH cohort, and superior sensitivity on the BH and UHB cohorts, which are the two more ethnically diverse cohorts.

**Table 1:** Equalized odds evaluation for ethnicity bias and COVID-19 status prediction test results across different models and test sets, optimized to sensitivities of 0.9

Test set	Model	EO (TP)	EO (FP)	Sensitivity	Specificity	PPV	NPV	F1	AUROC
PUH									
	RL	**0.047**^**a**^	0.037	0.876 (± 0.017)	0.512 (± 0.006)	0.088 (± 0.005)	0.987 (± 0.002)	0.159	0.834 (± 0.013)
	RL (unweighted)	**0.048**^**b**^	0.031	0.872 (± 0.017)	0.518 (± 0.006)	0.088 (± 0.005)	0.987 (± 0.002)	0.160	0.838 (± 0.013)
	ADV	0.050	**0.014**^**a**^	0.879 (± 0.017)	0.595 (± 0.005)	0.104 (± 0.005)	0.989 (± 0.001)	0.186	0.865 (± 0.012)
	NN	0.066	**0.028**^**b**^	0.890 (± 0.016)	0.631 (± 0.005)	0.114 (± 0.006)	0.991 (± 0.002)	0.202	0.875 (± 0.012)
	XGB	0.133	0.053	**0.919 (±** **0.013)**	0.532 (± 0.006)	0.095 (± 0.005)	**0.992 (±** **0.001)**	0.172	**0.882 (±** **0.011)**
	NN (weighted)	0.056	0.035	0.882 (± 0.017)	**0.650 (±** **0.005)**	**0.119 (±** **0.006)**	0.990 (± 0.002)	**0.210**	0.876 (± 0.012)
	XGB (weighted)	0.214	0.054	0.900 (± 0.015)	0.597 (± 0.006)	0.107 (± 0.006)	0.991 (± 0.002)	0.191	**0.882 (±** **0.012)**
UHB									
	RL	**0.057**^**a**^	**0.041**^**b**^	**0.879 (±** **0.034)**	0.574 (± 0.011)	0.079 (± 0.009)	0.991 (± 0.003)	0.144	0.849 (± 0.025)
	RL (unweighted)	0.155	0.044	0.876 (± 0.035)	0.538 (± 0.011)	0.073 (± 0.008)	0.991 (± 0.003)	0.135	0.834 (± 0.026)
	ADV	0.072	**0.039**^**a**^	0.867 (± 0.035)	0.637 (± 0.010)	0.090 (± 0.010)	0.991 (± 0.003)	0.163	0.865 (± 0.025)
	NN	**0.069**^**b**^	0.052	0.873 (± 0.035)	0.667 (± 0.010)	0.098 (± 0.011)	**0.992 (±** **0.002)**	0.176	**0.868 (±** **0.026)**
	XGB	0.106	0.071	0.867 (± 0.036)	0.585 (± 0.010)	0.080 (± 0.009)	0.991 (± 0.003)	0.146	0.854 (± 0.025)
	NN (weighted)	0.070	0.052	0.862 (± 0.037)	**0.725 (±** **0.010)**	**0.115 (±** **0.012)**	**0.992 (±** **0.002)**	**0.203**	0.866 (± 0.025)
	XGB (weighted)	0.155	0.042	0.867 (± 0.035)	0.603 (± 0.011)	0.083 (± 0.009)	0.991 (± 0.003)	0.151	0.858 (± 0.025)
BH									
	RL	**0.030**^**b**^	**0.010**^**a**^	**0.935 (±** **0.041)**	0.691 (± 0.029)	0.296 (± 0.043)	**0.987 (±** **0.008)**	0.449	**0.923 (±** **0.031)**
	RL (unweighted)	0.055	0.057	0.906 (± 0.049)	0.668 (± 0.029)	0.275 (± 0.031)	0.981 (± 0.011)	0.422	0.898 (± 0.035)
	ADV	**<0.001**^**a**^	0.045	0.877 (± 0.055)	0.779 (± 0.026)	0.356 (± 0.051)	0.979 (± 0.011)	0.506	0.912 (± 0.033)
	NN	0.059	0.039	0.870 (± 0.057)	0.803 (± 0.024)	0.380 (± 0.053)	0.978 (± 0.010)	0.529	0.912 (± 0.033)
	XGB	0.107	**0.036**^**b**^	0.928 (± 0.044)	0.644 (± 0.030)	0.266 (± 0.040)	0.985 (± 0.010)	0.413	0.908 (± 0.033)
	NN (weighted)	0.057	0.040	0.862 (± 0.057)	**0.834 (±** **0.023)**	**0.419 (±** **0.057)**	0.978 (± 0.010)	**0.564**	0.918 (± 0.032)
	XGB (weighted)	0.146	0.050	0.920 (± 0.045)	0.649 (± 0.030)	0.267 (± 0.040)	0.983 (± 0.010)	0.414	0.897 (± 0.036)

Equalized odds (EO) results are reported as the s.d. of true positive (TP) and false positive (FP) rates across all ethnicity labels, with bolded values denoting the best (^a^) and second best (^b^) scores. Classification metrics are reported alongside 95% CIs, with bolded values denoting best scores achieved on each test set. ADV, adversarial; XGB, XGBoost.

Although predictive performance of the RL model only varied slightly with respect to other models, the difference in accuracy of the RL model compared with that of other models was found to be statistically significant (*P* < 0.0001, by the Wilcoxon signed rank test).

In terms of fairness, the RL model achieved the best performance overall, achieving either the best or second-best equalized odds performances (for both true positive and false positive) across all external test cohorts, except for the false positive s.d. for PUH (Table 1). The adversarial model achieved the second best performance overall, usually achieving the best or second best scores for one of true positive or false positive s.d. metrics. The NN and XGBoost models with weights (inversely proportional to the frequency of sensitive attributes) were not found to improve equalized odds. In general, models with an added dynamic debiasing functionality (that is, RL or adversarial models) demonstrably improved equalized odds. Similar results were found when models were optimized to sensitivities of 0.85 (full numerical results in Supplementary Table 14), with RL generally achieving the best or second best equalized odds scores, demonstrating model consistency across small shifts in the decision threshold.

With the same goal of mitigating ethnicity biases, we additionally tested our method on a different classification task—patient discharge prediction. As before, we found that all models achieved reasonably high AUROC scores on the test set (Table 2), comparable to previously reported benchmarks using the same dataset^28^. AUROC scores ranged from 0.818 to 0.875 (CI: 0.805–0.886), with the XGBoost model achieving the highest score and the RL models (both with and without a debiasing component) achieving the lowest. However, when optimizing sensitivities to 0.9, RL (weighted) achieved the best results in terms of sensitivity and equalized odds, despite a small trade-off in AUROC. This was also the case when sensitivities were optimized to 0.85 (full numerical results in Supplementary Table 15), demonstrating model consistency. The difference in accuracy of the RL model compared with that of other models was found to be statistically significant (*P* < 0.0001, by the Wilcoxon signed rank test).

**Table 2:** Equalized odds evaluation for ethnicity bias and patient ICU discharge prediction test results across different models, optimized to sensitivities of 0.9

Model	EO (TP)	EO (FP)	Sensitivity	Specificity	PPV	NPV	F1	AUROC
RL	**0.032**^**a**^	**0.022**^**a**^	**0.897 (±** **0.015)**	0.539 (± 0.008)	0.171 (± 0.008)	0.980 (± 0.003)	0.287	0.829 (± 0.013)
RL (unweighted)	0.052	0.030	0.889 (± 0.016)	0.502 (± 0.008)	0.159 (± 0.008)	0.977 (± 0.003)	0.269	0.818 (± 0.013)
ADV	0.040	**0.027**	0.885 (± 0.016)	0.637 (± 0.008)	0.205 (± 0.010)	0.981 (± 0.003)	0.333	0.861 (± 0.012)
NN	**0.033**	0.037	0.884 (± 0.016)	0.600 (± 0.008)	0.189 (± 0.009)	0.980 (± 0.003)	0.312	0.847 (± 0.012)
XGB	0.062	**0.022**^**a**^	0.883 (± 0.016)	**0.674 (±** **0.008)**	**0.223 (±** **0.011)**	**0.982 (±** **0.003)**	**0.355**	**0.875 (±** **0.012)**
NN (weighted)	**0.032**^**a**^	0.033	0.882 (± 0.016)	0.606 (± 0.008)	0.191 (± 0.009)	0.980 (± 0.003)	0.314	0.843 (± 0.012)
XGB (weighted)	0.049	0.037	0.878 (± 0.016)	0.665 (± 0.007)	0.217 (± 0.010)	0.981 (± 0.003)	0.348	0.871 (± 0.011)

EO results are reported as the s.d. of TP and FP rates across all ethnicity labels, with bolded values denoting the best (^a^) scores. Classification metrics are reported alongside 95% CIs, with bolded values denoting best scores achieved on the test set.

### Debiasing hospital

**Fig. 3:**
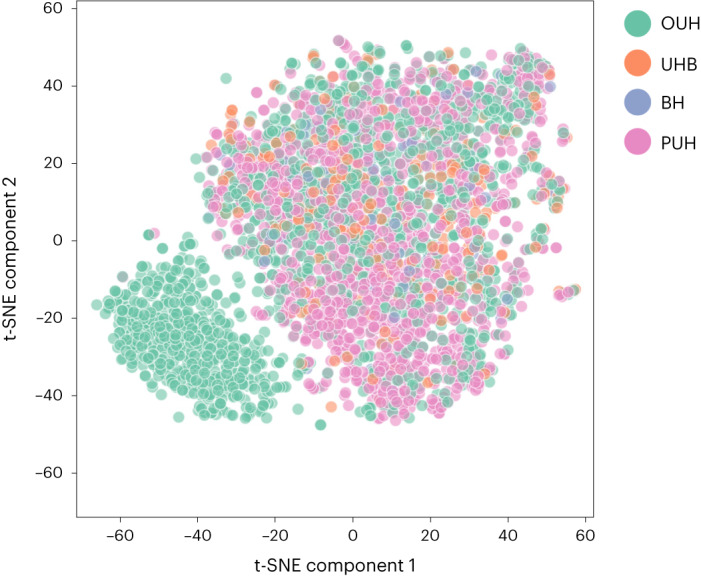
t-SNE representation. Visualization of clusters determined through a t-SNE, including all positive COVID-19 cases across four NHS trusts (OUH, PUH, UHB, BH).

We used *t*-stochastic neighbour embedding (t-SNE) to visualize a low-dimensional representation of all positive COVID-19 cases across the four NHS sites. From Fig. 3, we can see an isolated green cluster corresponding exclusively to a subset of presentations from OUH. This suggests that the training data can be clustered by, and thus is biased to, site-specific features such as annotation methods, data truncation, measuring devices or collection and processing tools. This was also found in a previous study using a different stratification of the same datasets^3^. These distribution shifts emphasize the importance of considering site-specific biases during model development.

For this bias-mitigation task, we tested all models on a held-out set, which included patient presentations from all four hospitals (Table 3). Model performances were higher than those achieved in the previous COVID-19 status prediction task (focused on ethnicity mitigation); and, as before, although performance was relatively consistent, the XGBoost and NN models (both weighted and unweighted) achieved the highest AUROC scores (AUROCs of 0.879 (CI: 0.865–0.892; RL), 0.855 (0.840–0.870; RL unweighted), 0.882 (0.869–0.896; adversarial), 0.891 (0.879–0.904; NN), 0.900 (0.887–0.912; XGBoost), 0.894 (0.881–0.906; NN weighted) and 0.901 (0.888–0.913; XGBoost weighted)). Using a sensitivity configuration of 0.9, we obtained consistent scores for sensitivity across all models (sensitivities of 0.887 (0.868–0.906; RL), 0.892 (0.873–0.911; RL unweighted), 0.882 (0.862–0.901; adversarial), 0.879 (0.859–0.898; NN), 0.875 (0.855–0.895; XGBoost), 0.883 (0.863–0.902; NN weighted) and 0.892 (0.873–0.911; XGBoost weighted)), with the weighted XGBoost and RL (unweighted) achieving the highest sensitivities and the weighted RL model achieving the third-highest sensitivity. As before, all models achieved high prevalence-dependent NPV scores (>0.985), demonstrating the ability to exclude COVID-19 with high confidence. Although the overall predictive performance between models was similar, the difference in accuracy of the RL model compared with that of other models was found to be statistically significant (*P* < 0.0001, by the Wilcoxon signed rank test).

**Table 3:** Equalized odds evaluation for hospital bias and COVID-19 status prediction test results across different models and test sets, optimized to sensitivities of 0.9

Model	EO (TP)	EO (FP)	Sensitivity	Specificity	PPV	NPV	F1	AUROC
RL	**0.010**^**a**^	**0.040**^**a**^	0.887 (± 0.019)	0.622 (± 0.008)	0.155 (± 0.009)	0.986 (± 0.003)	0.264	0.879 (± 0.014)
RL (unweighted)	0.035	0.063	**0.892 (±** **0.019)**	0.553 (± 0.008)	0.135 (± 0.008)	0.985 (± 0.003)	0.234	0.855 (± 0.015)
ADV	0.022	**0.045**^**b**^	0.882 (± 0.019)	0.642 (± 0.008)	0.161 (± 0.009)	0.986 (± 0.003)	0.272	0.882 (± 0.014)
NN	0.022	0.065	0.879 (± 0.019)	0.676 (± 0.008)	0.175 (± 0.010)	0.986 (± 0.002)	0.292	0.891 (± 0.013)
XGB	0.024	0.057	0.875 (± 0.020)	**0.720 (±** **0.008)**	**0.196 (±** **0.011)**	0.987 (± 0.003)	**0.320**	0.900 (± 0.012)
NN (weighted)	0.033	0.055	0.883 (± 0.019)	0.686 (± 0.008)	0.180 (± 0.011)	0.987 (± 0.002)	0.299	0.894 (± 0.013)
XGB (weighted)	**0.014**^**b**^	0.057	**0.892 (±** **0.019)**	0.681 (± 0.008)	0.179 (± 0.010)	**0.988 (±** **0.002)**	0.298	**0.901 (±** **0.012)**

EO results are reported as the s.d. of TP and FP rates across all hospital labels, with bolded values denoting the best (^a^) and second-best (^b^) scores. Classification metrics are reported alongside 95% CIs, with bolded values denoting best scores achieved on the test set.

In terms of bias mitigation, the proposed RL model (weighted) achieved the most fair performance, achieving the best result with respect to equalized odds (for both true positive and false positive s.d. scores). This result was consistent when models were optimized to sensitivities of 0.85 (full numerical results in Supplementary Table 16), demonstrating resilience of debiasing to decision thresholds. The adversarial model achieved the second-best performance in terms of false positive s.d., and the weighted XGBoost model achieved the second-best performance in terms of true positive s.d. Thus, as shown in the previous task, models with an added debiasing functionality demonstrably improved equalized odds, with only a slight trade-off in predictive performance (greatest AUROC decrease of 0.021 when comparing the RL model with the XGBoost implementation).

## Discussion

As ML gains more significance in clinical decision-making, it is crucial to ensure that these technologies neither reflect nor amplify undesirable biases. Incorporating fairness principles during model development and evaluation is essential to achieve this goal. Although our examination of variations across specific hospitals and ethnicities only addresses a portion of healthcare disparities, we aim to promote the utilization of deep RL and fairness principles in a wide range of prediction and debiasing tasks through the framework and concepts introduced.

For all tasks, we found that the outcomes of the RL models were less biased compared with those with no bias-mitigating component. However, although bias generally decreased, our models did not completely fulfil equalized odds requirements. One contributing factor may be that our training datasets, for all tasks, were imbalanced with respect to the sensitive attribute (that is, a much larger representation of white patients than other ethnicities; much more data was available from OUH and PUH than UHB and BH). As the base network we are using is an NN, skewed distributions can potentially give inconsistent results. This has previously been observed for adversarial training, as using balanced data was found to have a much stronger effect on fairness outcomes^20^. Thus, given sufficient data, future models could benefit from being trained on balanced datasets.

Because an RL set-up can help control how and when a learning signal is backpropagated to improve on error aggregation (which occurs through standard supervised learning), the use of cost-sensitive weighting (based on sensitive attribute frequency) may have been more effective for RL than when it was used in a standard supervised learning framework. In the standard supervised learning setting, cross-entropy loss provides a learning signal regardless of what is presented; thus, a model can become skewed or biased based on the majority class present in the batch. This was seen in the results for the weighted NN and XGBoost implementations, as equalized odds results were poorer. However, it should be noted that there may still be bias in the RL model given the population it is trained on, but this should not impact the performance on the test sets (which we have demonstrated temporally and externally). This bias may be a potential issue when transferring the model to a new domain, which can be an interesting area to explore in future studies.

With respect to fulfilling equalized odds requirements, the advantage of using an RL framework was more observable and clear (that is, noticeable improvements in true positive and false positive s.d. for RL results over other models) for the patient discharge task and the COVID-19 task that involved mitigating inter-hospital biases. This may be due to the larger amount of training data used in those tasks compared with the COVID-19 task with ethnicity debiasing (14,949 patients compared to 43,754 and 49,305 patients for COVID-19 ethnicity, COVID-19 hospital, and ICU patient discharge tasks, respectively). Having a greater amount of training data may have made it easier for models to confidently differentiate between different classes (for both the main task and the sensitive attribute). This was demonstrated by the COVID-19 tasks, as higher predictive performance was achieved for the hospital-site mitigation task than the ethnicity mitigation task, as the hospital-based task utilized a larger training set.

We employed threshold adjustment to ensure high sensitivity in our classification tasks, specifically for COVID-19 prediction and ICU patient discharge prediction. This technique is effective when dealing with imbalanced training data, which was the case for both tasks. However, we observed data bias due to site-specific factors in the t-SNE visualization (Fig. 3). Consequently, an optimal threshold derived from a specific dataset may not be suitable for new settings with different distributions. This probably contributed to the varying sensitivities observed between test sites in the COVID-19 task with ethnicity debiasing. Therefore, it is crucial to further investigate the selection of an optimal decision threshold, as it directly impacts classification and fairness metrics by affecting true positive and true negative rates. Additionally, in clinical settings, achieving consistent sensitivity (or specificity) scores across different sites is desirable, even if the AUROC remains consistent. Varying sensitivities and specificities can make it challenging for clinicians to rely on a model’s performance. Future experiments could explore the use of site-specific thresholds tailored during the calibration phase at each site to standardize predictive performance^19^. Moreover, while we recognize the usefulness of probability as a measurement, we chose classification for rapid triaging into ‘COVID-free’ or ‘COVID-suspected’ areas. However, depending on the task, probabilities can be used as the final outcome.

Furthermore, it is essential to consider the trade-off between sensitivity and specificity. In the COVID-19 task, we optimized thresholds for high sensitivity to aid in triaging. However, this trade-off negatively affected specificity. RL had high sensitivities but the lowest or second-lowest specificities. The trade-off should be carefully assessed for each task, as low specificity can strain hospitals because of increased resource utilization, follow-up tests, costs and patient anxiety or discomfort. This trade-off is also significant when determining fairness criteria. In certain tasks, high sensitivity may be preferred to minimize harm caused by false negatives, such as in disease diagnosis. In these cases, fairness metrics like true positive parity (equal opportunity) can be used, ensuring that the probability of the classifier predicting a sample as the positive class is equal across all classes of the sensitive attribute. It is important to note that other fairness metrics, such as statistical parity and test fairness, exist. Therefore, applications should be optimized with fairness definitions most suitable for each specific task.

While we demonstrated the effectiveness of our model in handling multi-class sensitive features, it remains crucial to consider whether a demographic-specific or site-specific model is more suitable compared to a generalized multi-class model for a given task. For instance, if the model’s purpose is to support patients within a specific hospital care structure or predict the risk of a disease known to vary significantly between ethnicities, utilizing personalized models trained individually on each class may be the optimal choice. However, implementing multiple models can be computationally demanding, posing challenges for hospitals. In such cases, adopting a more generalized model, like the debiasing framework presented here, would be advantageous, as it offers a feasible alternative while still addressing biases.

Understanding the complex interplay between genetic, social, and behavioural factors in clinical outcomes poses a significant challenge. While it is evident that ethnicity should not be the sole determining factor in certain non-clinical tasks like recidivism prediction, its role in clinical contexts is not always as straightforward. Ethnicity can be an important predictor for specific diagnoses, prognoses, and treatment recommendations^29^. In our COVID-19 screening task, we focused on addressing data imbalances to ensure fair predictions for minority groups using available data from UK hospital trusts. However, we acknowledge that ethnicity encompasses essential characteristics like place of residence and socioeconomic status, which collectively contribute to disease prevalence among specific ethnic groups. Defining the precise contribution of ethnicity (and related factors) to COVID-19 diagnosis during the early stages of a pandemic can be challenging. Nevertheless, as more data are collected over time, gradual adjustments should be made to accurately assess the true impact of these characteristics.

## Methods

Previous works have shown that ML-based methods can identify patients presenting with COVID-19 up to 90% sooner than polymerase chain reaction (PCR) testing, achieving high sensitivities and performing effectively as a rapid test-of-exclusion^3,19,27,30^. Additionally, one study showed that adversarial models were effective at screening for COVID-19 whilst being able to mitigate biases for selected sensitive features^3^. We aimed to build on these existing works, formulating a deep RL framework (with a specialized reward function) to effectively screen for COVID-19, while simultaneously mitigating unwanted biases.

### Datasets and preprocessing

To train and validate our models, we used clinical data with linked, deidentified demographic information for patients presenting to emergency departments across four independent UK NHS trusts: OUH, PUH, UHB and BH. With respect to these datasets, UK NHS approval via the national oversight and regulatory body, the Health Research Authority (HRA) through the Integrated Research Application System (IRAS), has been granted for development and validation of artificial intelligence models to detect COVID-19 (CURIAL; NHS HRA IRAS ID: 281832).

With scalability in mind, we trained models for the purposes of rapid triaging using laboratory blood tests and vital signs, as these are widely and routinely collected during the first hour of patients attending emergency care pathways in hospitals in middle- to high-income countries^30^. The features included are the same as those used in ref. ^19^ (also similar to those used in ref. ^27^ and ref. ^3^), allowing for comparison. Supplementary Table 2 summarizes the final features used.

For each of the models, a training set was used for model development, hyperparameter selection and model training; a validation set was used for continuous validation and threshold adjustment; and, after successful development and training, three held-out, external test sets were used to evaluate the performance of the final models.

For the training and validation sets used in the ethnicity debiasing models, we used patient presentations exclusively from OUH. From OUH, we curated two data extracts—one from the first wave of the COVID-19 pandemic in the UK (1 December 2019 to 30 June 2020) and one from the second wave (1 October 2020 to 6 March 2021) (Supplementary Fig. 1). Owing to incomplete penetrance of testing during the first wave and imperfect sensitivity of the PCR test, there is uncertainty in the viral status of patients presenting who were untested or tested negative. Thus, from the ‘wave one’ dataset, we only included the positive cases (as determined through PCR tests) in training; and from the ‘wave two’ dataset, we included both positive COVID-19 cases (by PCR) and negative controls. This was done to ensure that the label of COVID-19 status was correct during training. This resulted in a prevalence of 11.1% used during training, which is within the spatial and temporal range of prevalences observed across the UK trusts used in our study (prevalences between 4.27–12.2%; Supplementary Table 1). Furthermore, to reasonably evaluate classification performance with respect to ethnicity, we removed any presentations where the label for ethnicity was ambiguous, including those labelled as ‘unknown’, ‘mixed’ or ‘other’. This resulted in 18,687 patients used in training and validation, including 2,083 of which were COVID-19 positive. A ratio of 80:20 was used to split the OUH cohort into training and validation sets. We then performed external validation on three independent patient cohorts from PUH, UHB and BH (totalling 38,964 admitted patients, including 1,963 who were COVID-19 positive). From Supplementary Table 3, we can see that ethnicity is heavily skewed in our training dataset, making it a possible source of bias during training.

We performed sensitivity analysis to account for uncertainty in the viral status of patients testing negative by PCR or who were not tested. We evaluated this on the validation set (to ensure that the test sets were not used until a final model is developed), achieving AUROC scores of 0.836 (0.811–0.860) and 0.857 (0.833–0.880) for the original and adjusted training sets, respectively. The comparable results (overlapping CIs) demonstrate model stability across the training sets.

In addition to debiasing ethnicity, we also demonstrated the utility of our proposed method for debiasing with respect to the hospital that a patient attended. To evaluate bias related to hospital location, presentations from multiple sites needed to be present in the training data. Thus, we combined presentations from all hospital cohorts previously described; however, we additionally included the patient presentations with ambiguous ethnicity labels, as we are no longer focusing on mitigating ethnicity-based biases. Using a 60:20:20 split, we separated the data into training, validation and test sets, respectively, resulting in 58,339 presentations used in training and validation (including 4,245 that were COVID-19 positive) and 14,585 presentations in the held-out test set (including 1,056 that were COVID-19 positive).

Consistent with previous studies, we addressed the presence of missing values by using population median imputation, then standardized all features in our data to have a mean of 0 and an s.d. of 1.

A summary of training, validation and test cohorts used in each task can be found in Supplementary Tables 3 and 4. The full inclusion and exclusion criteria for patient cohorts and summary population statistics can be found in Supplementary Section C.

To further test our framework, we also trained models to predict the discharge status of patients staying in the ICU, using data from the eICU Collaborative Research Database (eICU-CRD)^31,32^. The eICU-CRD is a publicly available, anonymized database with pre-existing institutional review board approval. The database is released under the Health Insurance Portability and Accountability Act safe harbour provision. The re-identification risk was certified as meeting safe harbour standards by Privacert (Health Insurance Portability and Accountability Act certification no. 1031219-2). Here, we also focus on debiasing ethnicity (which, again, is heavily imbalanced in the dataset), demonstrating the generalizability of our method on a new, independent clinical task. Details on the dataset, features and preprocessing steps used for this task can be found in Supplementary Section D.

### Reinforcement learning for classification

To formulate classification as an RL task, we model our problem in a sequential decision-making format using a finite Markov decision process. We define the Markov decision process using a tuple of five variables (*s*, *a*, *p*, *r*, *γ*), where: *s* is the state space of the process, *a* is the action that an agent takes, *p* is the transition probability that results from an action, *r* is the reward expected for a given action and *γ* is the discount factor used for future rewards.

For a given *N* × *D* dataset, *N* is the total number of samples and *D* is the number of features in each sample. During training, a batch of data is randomly shuffled and presented to the model in order. Here, *P* is deterministic, as the agent moves from one state to the next according to the order of samples in the training data. The features of each sample presented makes up the state, *s*.

The action, *a*, is the prediction the agent makes when presented with a state, *s*. Given a total number of classification labels, *K*, each *a* is selected from one of *K* classes. With respect to COVID-19 classification, *a* ∈ {0, 1}, where 0 corresponds to COVID-19 negative cases and 1 corresponds to COVID-19 positive cases.

Because the selection of an action, *a*, does not determine the following sample, *s*, presented to the agent, an alternative dependency must be introduced between *s* and *a*. To achieve this, a training episode is terminated when an agent incorrectly classifies the minority class, preventing any further reward, *r*. This allows for a relationship between *s* and *a* to be learned, especially when there are severe data imbalances between majority (COVID-19 negative) and minority (COVID-19 positive) classes^26^. We have specifically chosen to use this off-policy Monte Carlo (that is model-free) RL approach, as an off-policy algorithm allows for the samples presented to the network to be independent and uncorrelated; and the model-free element means we don’t learn a transition function (and thereby don’t learn a trajectory), but instead, learn a mapping of state to appropriate action for all considered states. Additionally, the temporal difference loss allows us to estimate the equivalent Monte Carlo return in a more efficient manner^33^, thus making it feasible to treat each state independently. The overall RL framework is shown in Fig. 1.

#### Defining reward for bias mitigation

Standard classification models that use gradient descent, estimate the marginal distribution with a differentiable error term. This can skew models towards the majority class present in a batch, due to aggregation of the errors. However, RL provides a way of indicating error using a non-differentiable signal that can be uniquely designed for each situation at hand; for example, for our purposes, we can detect minority classes by representing this in the reward function, which aggregation typically doesn’t allow you to do. As a result, an RL paradigm allows for the learning of minority classes without needing to compromise on learning of majority classes, implicitly. This is particularly important in the tasks presented here, where we aim to train models that can generalize well across different patient demographics, patient outcomes and hospital centres, even if their distributions are unequal at the time of model development.

The reward, *r*, is the signal evaluating the success of the agent’s selected action. We introduce a specialized function for reward, uniquely formulated for the purpose of mitigating biases of the chosen sensitive feature, *z*. To do this, we separate the reward function into two components—one to help train a strong classifier and one to debias with respect to the sensitive attribute. Additionally, as the majority of previous studies have exclusively evaluated bias mitigation for binary attributes, we formulated the reward function to be able to debias multi-class attributes. This is especially important in clinical tasks, as a higher degree of granularity is often required since binning values to fewer (that is, binary) classes may not be biologically relevant (especially when classes are categorical) and is heavily biased on the sample population^3,26^. Thus, to accommodate for class imbalance for multi-class sensitive features, we make the reward inversely proportional to the relative presence of a class in the data. This is comparable to using cost-sensitive weights in standard supervised learning. While cost-adjusted weights can help address class imbalances, they still rely on the cross-entropy loss, which provides the network with a learning signal regardless of what is presented to it; thus, skewing models towards the majority class present in a batch, due to aggregation of the errors. By instead implementing an RL set-up (rather than a supervised learning framework dependent on gradient descent), one can control how and when a learning signal is backpropagated (further explanation in ‘Double deep *Q*-learning’ and ‘Reinforcement learning training procedure’). This has been demonstrated in previous studies on imbalanced learning (with respect to the outcome class label), where RL (with a specialized reward function) was compared with other common imbalanced learning methods (Synthetic Minority Oversampling Technique (SMOTE), cost-sensitive and cost-adjusted weights), and found to improve on balanced classification^25,26^.

During model training, a positive reward is given when the agent correctly classifies the sample (as either COVID-19 positive or negative) and a negative reward is given otherwise. If a negative reward is given (that is, a sample was misclassified), the absolute reward value given is inversely proportional to the relative presence of the label of the sample in the training data. Thus, the absolute reward value of a sample from the minority class is higher than that in the majority class, making the model more sensitive to the minority class. This helps accommodate for label imbalance during training; and since the primary purpose of the model is to effectively classify COVID-19 status, this sensitivity differential will help the agent learn the optimal behaviour for COVID-19 prediction. To consider the sensitive class, *z* (which we aim to debias), we make the absolute reward for the positive case (that is, when a sample is correctly classified) inversely proportional to the relative presence of each respective *z* label in the training data, accommodating for any class imbalances present in a multi-class sensitive attribute. Here, the absolute reward value of a sample from a minority *z* class is therefore higher than that from the majority class, making the model more sensitive to minority *z* labels. By performing debiasing on the positively rewarded states, we already know that the sample was correctly classified for the main task, as debiasing would be inconsequential if the model was not clinically effective for use to begin with. The formulation introduced allows for evaluation of both binary and multi-class tasks and sensitive features. Assuming *M* classes in *z*, and using *l*_*k*_ to represent the COVID-19 label, the reward function is formulated as follows:

1 $$R({s}_{t},{a}_{k},{l}_{k})=\left\{\begin{array}{ll}{\lambda }_{m}\quad &\,{{\mbox{if}}}\,\,{a}_{k}={l}_{k}\\ -{\lambda }_{k}\quad &\,{{\mbox{if}}}\,\,{a}_{k}\ne {l}_{k}\end{array}\right.$$

2 $${\lambda }_{{{{{k}}}}}=\frac{\frac{1}{{N}_{k}}}{{\left\Vert \frac{1}{{N}_{0}},\frac{1}{{N}_{1}},\ldots ,\frac{1}{{N}_{k}}\right\Vert }^{2}}$$

3 $${\lambda }_{{{{{m}}}}}=\frac{\frac{1}{{N}_{m}}}{{\left\Vert \frac{1}{{N}_{0}},\frac{1}{{N}_{1}},\ldots ,\frac{1}{{N}_{m}}\right\Vert }^{2}}$$

*N* represents the number of instances in class *k* or *m*, and *λ* is a trade-off parameter used for adjusting the influence of each class. We found that models achieved desirable performance when *λ* is the vector-normalized reciprocal of the number of class instances, as shown in equations (2) and (3). To balance immediate and future rewards, a discount factor, *γ* = 0.1, is used.

#### Policy iteration by *Q*-learning

An optimal policy π* is one that maximizes the expected cumulative reward, *v*^*π*^. This can be interpreted as the value of a state-action combination, and is discovered by iterating through a series of policies, ${\{\pi \}}_{i}^{k}$, where π* = *π*^*k*^. Using the Bellman equation, *v*^*π*^ can be determined by solving a system of linear equations and calculating a set of *Q*-values, where *Q* represents the action-value function:

4 $${Q}_{i}^{\pi }({s}_{t},{a}_{t})=r({s}_{t},{a}_{t})+\gamma \mathop{\sum}\limits_{{s}_{t+1}}p({s}_{t+1}| {s}_{t},{a}_{t}){v}_{i}^{\pi }({s}_{t+1}),$$

This gives successive policies:

5 $${\pi }_{i+1}({a}_{t}| {s}_{t})={\rm{arg}}\mathop{\max }\limits_{a}{Q}_{i}^{\pi }({s}_{t},{a}_{t}),$$

where, ${\pi }^{* }={\rm{arg}}\mathop{\max }\limits_{a}{Q}^{* }$. Finally, we can use the advantage function, *A*^*π*^, to relate the state-action value function and *Q* function:

6 $${A}^{\pi }({s}_{t},{a}_{t})={Q}^{\pi }({s}_{t},{a}_{t})-{V}^{\pi }({s}_{t})$$

The value function, *V*^*π*^ can be viewed as a proxy for the ‘goodness’ of a particular state, and the *Q*^*π*^ function evaluates the value of selecting a particular action in this state^34^. Thus, *A*^*π*^ can be interpreted as the relative importance of each action.

#### Duelling *Q*-network architecture

In a typical deep *Q*-network (DQN) set-up, the output layer of the network corresponds to predicted *Q*-values for state-action pairs. Since only one state-action pair can be trained at a time, it can be difficult to provide update information about the state. To address this, we implement a duelling *Q*-network, which is capable of training state representations and action representations independent of one another.

For a DQN, the *Q*-network is implemented as a standard, single-stream NN, where fully connected layers are connected in a continuous sequence. The duelling *Q*-network (Fig. 2), instead, implements a fully connected NN with two streams—one for estimating the value (which is scalar) and another to estimate the advantages of each action (which is a vector). These two streams are combined to produce a single output, which is the *Q-*function^34^.

Based on the definition of the advantage function, we represent *Q* as:

7 $$\begin{array}{l}{Q}_{i}^{\pi }({s}_{t},{a}_{t};{\theta }_{i},{\alpha }_{i},{\beta }_{i})\\={V}_{i}^{\pi }({s}_{t};{\theta }_{i},{\beta }_{i})+\left({A}_{i}^{\pi }({s}_{t},{a}_{t};{\theta }_{i},{\alpha }_{i})-{{{\rm{softmax}}}}({A}_{i}^{\pi }({s}_{t},{a}_{t+1};{\theta }_{i},{\alpha }_{i}))\right),\end{array}$$

where *α* and *β* represent the parameters of the *A* and *V* streams of the fully connected layers, respectively. The additional softmax module is to allow *Q* to recover *V* and *A* uniquely^34^. Additionally, this extra term does not change the relative rank of *A* (and subsequently, *Q*-values), which preserves the *ϵ*-greedy policy (which we use in our training; explained in ‘Reinforcement learning training procedure’).

For the *Q*-network, we used a fully connected NN with one hidden layer, alongside the rectified linear unit (ReLU) activation function and dropout. For updating model weights, the Adam (Adaptive Moment Estimation) optimizer was used during training. We set the exploration probability, *ϵ*, to be linearly attenuated from 1 to 0.01 over the entire training process. Each training period consists of 120,000 steps (that is, iterations of updating parameters *θ*).

#### Double deep *Q*-learning

During each episode, combinations of states, actions, and rewards at each step, (*s*_*t*_, *a*_*t*_, *r*_*t*_, *s*_*t*+1_), are saved in the agent’s working memory, *M*. To learn the parameters of the *Q*-network, *θ*, a randomly sampled subset of these transitions, *B*, are used in the gradient descent step. The mean-squared error loss function is used to optimize the network:

8 $$L({\theta }_{i})=\mathop{\sum}\limits_{({s}_{t},{a}_{t},{r}_{t},{s}_{t+1})\in B}{(y-Q({s}_{t},{a}_{t};{\theta }_{i}))}^{2}$$

As in standard supervised learning, *y* can be treated as the target to be predicted and *Q*(*s*, *a*; *θ*_*i*_) as the prediction. We define *y* using the format of a DDQN.

As a standard DQN uses the current *Q*-network to determine an action, as well as estimate its value, it has been shown to give overoptimistic value estimates^35^. This increases the likelihood of selecting overestimated values (which can occur even when action values are incorrect), making it harder to learn the optimal policy. Thus, DDQN was introduced as a method of reducing this overestimation^36^. Unlike a DQN, a DDQN uses the current *Q*-network to select actions, and the target *Q*-network to estimate its value^37^. Through decoupling the selection and evaluation steps, a separate set of weights, ${\theta }^{{\prime} }$, can be used to provide an unbiased estimate of value.

The DDQN algorithm is implemented using the following target function:

9 $${y}_{i}={r}_{t}+(1-{\rm{term}})\gamma Q\left({s}_{t+1},{\rm{arg}}\mathop{\max }\limits_{a}Q({s}_{t+1},{a}_{t+1};{\theta }_{i});{\theta }_{i}^{{\prime} }\right)$$

As previously mentioned, a dependency between a state and action needs to be established for the agent to learn a relationship. Thus, within this function, the value of ‘term’ is set to 1 once the agent reaches its terminal state, and 0 otherwise. A terminal state is reached after the agent has iterated through all samples in the training data (or a set number of samples, specified at the beginning of training), or when the agent misclassifies a sample from the minority class (preventing any further reward).

### Reinforcement learning training procedure

As a typical supervised learning model relies on standard cross-entropy loss, a network is provided with a learning signal regardless of what is presented to it. However, by framing the learning problem as an RL set-up, learning can be regulated through the design of the reward function. This allows for one to control how and when a learning signal is backpropagated (recall the ‘term’ variable defined), which aggregation (through standard supervised learning) typically doesn’t allow for.

The environment reward procedure is described in Algorithm 1. The overall *Q*-network is trained according to the DDQN process described in Algorithm 2. The final, optimized *Q*-network is considered to be the trained classifier. In each episode, the agent employs an *ϵ*-greedy behaviour policy to select an action, which randomly selects an action with probability *ϵ*, or an action following the optimal Q function, ${\rm{arg}}\mathop{\max }\nolimits_{a}{Q}^{* }({s}_{t},{a}_{t})$ with probability 1 − *ϵ*.

#### Algorithm 1 Environment reward procedure



#### Algorithm 2 DDQN training procedure



### Model comparators and evaluation metrics

To assess the effectiveness of our method, we compare it against two baseline model architectures—a fully connected NN and XGBoost. Additionally, we compare our method to an adversarial debiasing framework, which currently represents the state-of-the-art approach for addressing algorithmic biases. XGBoost is a widely used ensemble model known for achieving state-of-the-art performance in various ML challenges. The fully connected NN serves as the foundation for both our RL-based framework and an adversarial debiasing framework. Furthermore, we evaluate our method against an RL classification model without any debiasing component (specifically, an implementation introduced previously^26^). This comparison ensures that our model is initially trained as a strong classifier and that the inclusion of a debiasing component is indeed beneficial.To account for the imbalanced distribution of sensitive attribute labels, we employ cost-adjusted weighting within the RL framework. To have a fair comparison, we also train implementations of the NN and XGBoost models using the same weighting strategy.All comparator methods have previously been shown to be able to effectively screen for COVID-19, using the same datasets, allowing for direct comparison of our method^3,26,27,30^. Details on network architectures of comparator models can be found in Supplementary Section B.To evaluate the performance of COVID-19 prediction, we report sensitivity, specificity, positive and negative predictive values (PPV and NPV), and the AUROC, alongside 95% CIs based on standard error. CIs for AUROC are calculated using Hanley and McNeil’s method. The Wilcoxin signed rank test is used to calculate *P-*values, comparing the difference in accuracy between different models.As the purpose of our framework is to train models that are unbiased towards sensitive features, *z*, we evaluate model fairness using the statistical definition of equalized odds. Here, a classifier is considered fair if true positive rates are equal and false positive rates are equal, across all possible classes of the sensitive attribute^7^. To assess multiple labels (that is, >2), we used the s.d. of true positive and false positive scores^3^. Standard deviation scores closer to zero suggest greater outcome fairness. The equations used to calculate true positive and false positive s.d. scores are as follows:

10 $$\begin{array}{rcl}{\rm{s.d.}}_{\rm{TP}}&=&{\rm{s.d.}}\left(\left\{P(\hat{Y}=1| Y=1,Z={z}_{i}),P(\hat{Y}=1| Y=1,Z={z}_{i+1}),\right.\right.\\ &&\left.\left.\ldots ,P(\hat{Y}=1| Y=1,Z={z}_{N})\right\}\right)\\ &=&{\rm{s.d.}}\left(\left\{\frac{{\rm{TP}}_{i}}{{\rm{TP}}_{i}+{\rm{FN}}_{i}},\frac{{\rm{TP}}_{i+1}}{{\rm{TP}}_{i+1}+{\rm{FN}}_{i+1}},\ldots ,\frac{{\rm{TP}}_{N}}{{\rm{TP}}_{N}+{\rm{FN}}_{N}}\right\}\right),\end{array}$$

11 $$\begin{array}{rcl}{\rm{s.d.}}_{\rm{FP}}&=&{\rm{s.d.}}\left(\left\{P(\hat{Y}=1| Y=0,Z={z}_{i}),P(\hat{Y}=1| Y=0,Z={z}_{i+1}),\right.\right.\\ &&\left.\left.\ldots ,P(\hat{Y}=1| Y=0,Z={z}_{N})\right\}\right)\\ &=&{\rm{s.d.}}\left(\left\{\frac{{\rm{FP}}_{i}}{{\rm{TP}}_{i}+{\rm{FN}}_{i}},\frac{{\rm{FP}}_{i+1}}{{\rm{TP}}_{i+1}+{\rm{FN}}_{i+1}},\ldots ,\frac{{\rm{FP}}_{N}}{{\rm{TP}}_{N}+{\rm{FN}}_{N}}\right\}\right)\end{array}$$

### Hyperparameter optimization and threshold adjustment

In each task, appropriate hyperparameter values were determined through grid search and standard fivefold cross-validation, using respective training sets. Grid search was used to determine: (1) the number of nodes to be used in each layer of the NN and RL models, (2) the learning rate and (3) the dropout rate. Fivefold cross-validation was used to ensure that hyperparameter values were evaluated on as much data as possible, as to provide the best estimate of potential model performance on new, unseen data. Details on the hyperparameter values used in the final models can be found in Supplementary Section E.

The raw output of many ML classification algorithms is a probability of class membership, which is then mapped to a particular class. For binary classification, the default threshold is typically 0.5, where values equal to or greater than 0.5 are mapped to one class and all other values are mapped to the other. However, this default threshold can lead to poor sensitivity, especially when there is a large class imbalance (as seen with our training datasets, where there are far more COVID-19 negative cases than positive ones). Thus, we used a grid search to adjust the decision boundary used for identifying COVID-19 positive or negative cases, to improve detection rates at the time of testing. For our purposes, the threshold was optimized to sensitivities of 0.9 to ensure clinically acceptable performance in detecting positive COVID-19 cases. This sensitivity was chosen to exceed the sensitivity of lateral flow device tests, which achieved a sensitivity of 56.9% (95% CI: 51.7–62.0%) for OUH admissions between 23 December 2021 and 6 March 2021^28^. Additionally, the gold standard for diagnosing viral genome targets is by real-time PCR, which has estimated sensitivities of approximately 80–90%^38,39^. Therefore, optimizing to a threshold of 0.9 will ensure that models can effectively detect COVID-19 positive cases and exceed the sensitivities of current diagnostic testing methods.

We chose to represent this task as a binary classification (COVID-19 positive or negative) rather than a probability score, to correspond to the green–amber–blue categorization system adopted by trust policy, with green representing a patient whose illness has no features of COVID-19, amber representing an illness with features potentially characteristic with COVID-19 and blue representing laboratory-confirmed COVID-19 infection. Thus, having a classification result is consistent with rapid triage into a green or amber pathway.

### Reporting summary

Further information on research design is available in the Nature Portfolio Reporting Summary linked to this article.

## Supplementary information

Supplementary Information Supplementary Fig. 1, Tables 1–16 and text.

Reporting Summary

## Data Availability

Data from OUH studied here are available from the Infections in Oxfordshire Research Database (https://oxfordbrc.nihr.ac.uk/research-themes/modernising-medical-microbiology-and-big-infection-diagnostics/infections-in-oxfordshire-research-database-iord/), subject to an application meeting the ethical and governance requirements of the database. Data from UHB, PUH and BH are available on reasonable request from the respective trusts, subject to HRA requirements. The eICU Collaborative Research Database is available online at https://www.physionet.org/content/eicu-crd/2.0/.

## Code availability

Code for this publication can be found at https://codeocean.com/capsule/6724009 (ref. ^40^) and https://github.com/yangjenny/BiasMitigationRL (ref. ^41^).
