# Supplementary material for: Algorithmic fairness and bias mitigation for clinical machine learning with deep reinforcement learning
Source: Nat Mach Intell. 2023 Jul 31;5(8):884–94. doi: 10.1038/s42256-023-00697-3 (PMC10442224; doi:10.1038/s42256-023-00697-3)
Supplement: Supplementary file 1 — Supplementary Fig. 1, Tables 1–16 and text. [file 42256_2023_697_MOESM1_ESM.pdf]

nature machine intelligence

Article

https://doi.org/10.1038/s42256-023-00697-3

Algorithmic fairness and bias mitigation for clinical machine learning with deep reinforcement learning

In the format provided by the authors and unedited

nature machine intelligence

Article

https://doi.org/10.1038/s42256-023-00697-3

Algorithmic fairness and bias mitigation for clinical machine learning with deep reinforcement learning

In the format provided by the
authors and unedited

Supplementary Material

Table of Contents

A. Software Packages and Implementation

B. Model Architectures

C. COVID-19 Data and Preprocessing

D. eICU-CRD Data and Preprocessing

E. Hyperparameter Values

F. Threshold Values

G. Additional Results

A Software Packages and Implementation

Models were implemented using Python (v3.6.9). Scikit Learn (v0.24.1) was used for standardization, median imputation, and calculating performance metrics. Performance metrics were calculated using Scikit Learn and manually programmed. XGBoost baseline models were implemented using the XGBoost library (v1.3.3). Neural network baseline models were implemented using Keras (v2.6.0). Reinforcement learning was set up using Tensorflow (v2.6.2). All models were run using an Intel Xeon E-2146G Processor (CPU: 6 cores, 4.50 GHz max frequency). The code for this paper is available online at `https://github.com/yangjenny/BiasMitigationRL` and `https://codeocean.com/capsule/6724009`.

B Model Architectures

Neural Network Model: The rectified linear unit (ReLU) activation function was used for the hidden layers and the sigmoid activation function was used in the output layer. For updating model weights, the Adaptive Moment Estimation (Adam) optimizer was used during training.

XGBoost Model: XGBoost is a popular ensemble model that has achieved state-of-the-art results on many machine learning challenges. Ensemble methods combine the predictions of multiple models, such that the generalization error is improves (i.e., contribution of individual error from any individual model is lessened). XGBoost in particular, utilizes a boosting technique, where trees are sequentially added and fit to correct for the prediction errors made by previous models. Default settings were used in all experiments.

Adversarial Debiasing Model: The adversarial debiasing architecture used consists of two individual networks – a predictor network, P, and an adversary network, A. P and A are each a multilayer perceptron (MLP). Here, P is trained to predict COVID-19 status (or patient ICU discharge status), given a set of clinical features. Its raw output, $\hat{y}$ – the predicted probability score, and the true label, y, are then used as the input to A, which tries to predict z. This is because we look at evaluating equalized odds, which considers $\hat{y}$ and y. However, depending on the definition of fairness being achieved, the adversary may have other inputs (e.g. only $\hat{y}$ if evaluating demographic parity) [3,6]. For our purposes, z is either hospital site or ethnicity. We use cross-entropy loss (and binary cross-entropy loss when the feature is binary), where L_P represents the loss for P , and L_A represents the loss for A. And as introduced in [6], the loss function for P is:

$$L = L_P + \frac{L_P}{L_A} - \alpha L_A \tag{1}$$

For P, the sigmoid activation function is used in the output layer; and for A, the softmax activation function is used instead.

We use the implementation from: `https://github.com/yangjenny/adversarial_learning_bias_mitigation`

C COVID-19 Data and Preprocessing

Oxford University Hospitals NHS Foundation Trust (OUH): We included all patients attending acute and emergency care settings at OUH who received routine blood tests on arrival, considering presentations before December 1, 2019, and thus before the pandemic, as the COVID-19-negative (control) cohort. We considered presentations during the 'first wave' of the UK COVID-19 pandemic (December 1, 2019 to June 30, 2020) with PCR confirmed SARS-CoV-2 infection as the COVID-19-positive (cases) cohort. We excluded patients who opted out of electronic health record (EHR) research and those who did not receive laboratory blood tests or were younger than 18 years of age. Due to incomplete penetrance of testing during the first wave of the pandemic, and imperfect sensitivity of the PCR test, there is uncertainty in the viral status of patients presenting during the pandemic who were untested or tested negative. We therefore selected a pre-pandemic control cohort during training to ensure absence of disease in patients labelled as COVID-19-negative. Clinical features extracted for each presentation included first-performed blood tests, blood gases, vital signs measurements and PCR testing for SARS-CoV-2 (Abbott Architect [Abbott, Maidenhead, UK], TaqPath [Thermo Fisher Scientific, Massachusetts, USA] and Public Health England-designed RNA-dependent RNA polymerase assays).

Portsmouth Hospitals University NHS Foundation Trust (PUH): PUH considered all patients admitted to the Queen Alexandria Hospital, serving a population of 675,000 and offering tertiary referral services to the surrounding region, between March 1, 2020 and February 28, 2021. Confirmatory COVID-19 testing was by laboratory SARS-CoV2 RT-PCR assay, considering any positive PCR result within 48hrs of admission as a true positive.

University Hospitals Birmingham NHS Foundation Trust (UHB): UHB considered all patients admitted to The Queen Elizabeth Hospital, Birmingham, between December 01, 2019 and October 29, 2020. The Queen Elizabeth Hospital is a large tertiary referral unit within the UHB group which provides healthcare services for a population of 2.2 million across the West Midlands. Confirmatory COVID-19 testing was performed by laboratory SARS-CoV-2 RT-PCR assay.

Bedfordshire NHS Foundation Trust (BH): BH considered all patients admitted to Bedford Hospital between January 1, 2021 and March 31, 2021. BH provides healthcare services for a population of around 620,000 in Bedfordshire. Confirmatory COVID-19 testing was performed on the day of admission by point-of-care PCR based nucleic acid testing [SAMBA-II & Panther Fusion System, Diagnostics in the Real World, UK, and Hologic, USA].

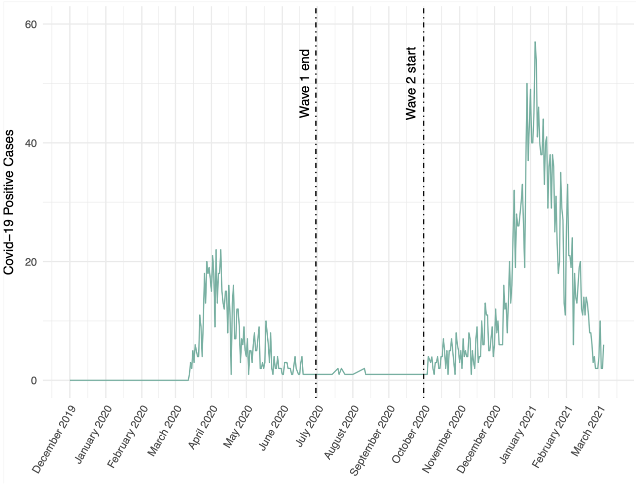

Supplementary Figure 1: Plot of OUH positive cases, showing the first "wave" of the COVID-19 epidemic in the UK from December 1, 2019 to June 30, 2020; and the second "wave" from October 1, 2020 – March 6, 2021.

Supplementary Table 1: Summary population characteristics for OUH training cohorts, prospective validation cohort of patients attending OUH, independent validation cohorts of patients admitted to three independent NHS Trusts. *indicates merging for statistical disclosure control.

	OUH (wave 1 cases)	OUH (wave 2 cases)	PUH	UHB	BH
***n*, patients**	701	22,857	37,896	10,293	1177
***n*, COVID positive**	701	2,012 (8.80%)	2,005 (5.29%)	439 (4.27%)	144 (12.2%)
**Sex:**					
- Male (%)	376 (53.64)	11409 (49.91)	20839 (54.99)	4831 (46.93)	627 (53.27)
- Female (%)	325 (46.36)	11448 (50.09)	17054 (45.0)	5462 (53.07)	549 (46.64)
Age, yr (IQR)	72 (55-82)	67 (49-80)	69 (48-82)	63 (42-79)	68.0 (48-82)
**Ethnicity:**					
-White (%)	480 (68.47)	17387 (76.07)	28704 (75.74)	6848 (66.53)	1024 (87.0)
-Not Stated (%)	128 (18.26)	4127 (18.06)	8389 (22.14)	1061 (10.31)	≤10
-South Asian (%)	22 (3.14)	441 (1.93)	170 (0.45)	1357 (13.18)	71 (6.03)
-Chinese (%)	*	51 (0.22)	42 (0.11)	41 (0.4)	≤10
-Black (%)	25 (3.57)	279 (1.22)	187 (0.49)	484 (4.7)	36 (3.06)
-Other (%)	34 (4.85)*	410 (1.79)	269 (0.71)	333 (3.24)	29 (2.46)
-Mixed (%)	12 (1.71)	162 (0.71)	135 (0.36)	169 (1.64)	13 (1.1)

Supplementary Table 2: Clinical predictors considered for COVID-19 status prediction.

**Category**	**Features**
Vital Signs	Heart rate, respiratory rate, systolic blood pressure, diastolic blood pressure, temperature
Blood Tests	Haemoglobin, haematocrit, mean cell volume, white cell count, neutrophil count, lymphocyte count, monocyte count, eosinophil count, basophil count, platelets
Liver Function Tests & C-reactive protein	Albumin, alkaline phosphatase, alanine aminotransferase, bilirubin, C-reactive protein
Urea & Electrolytes	Sodium, potassium, creatinine, urea, estimated glomerular filtration rate

Supplementary Table 3: Summary of number of patients, COVID-19 positive cases, and ethnicity distribution for training, validation, and external test set cohorts included in the ethnicity debiasing task.

	Training	Validation	External Test		
	OUH	OUH	PUH	UHB	BH
***n*, patients**	14,949	3,738	29,103	8,730	1,131
***n*, COVID-19 positive (PCR)**	1,672	411	1,478	347	138
**Ethnicity:**					
White (%)	14,293 (95.6)	3,574 (95.6)	28,704 (98.6)	6,848 (78.4)	1,024 (90.5)
South Asian (%)	370 (2.5)	93 (2.5)	170 (0.6)	1357 (15.5)	71 (6.3)
Black (%)	243 (1.6)	61 (1.6)	187 (0.6)	484 (5.5)	36 (3.2)
Chinese (%)	43 (0.3)	10 (0.3)	42 (0.1)	41 (0.5)	0 (0.0)

Supplementary Table 4: Summary of number of patients, COVID-19 positive cases, and hospital case distribution for training, validation, and held-out test set cohorts used in hospital debiasing task.

	Training	Validation	Test
***n*, patients**	43,754	14,585	14,585
***n*, COVID-19 positive (PCR)**	3,171	1,074	1,056
**Hospital:**			
OUH (%)	14,104 (32.2)	4,694 (32.2)	4,760 (32.6)
PUH (%)	22,750 (52.0)	7,596 (52.1)	7,550 (51.8)
UHB (%)	6,186 (14.1)	2,062 (14.1)	2,045 (14.0)
BH (%)	714 (1.6)	233 (1.6)	230 (1.6)

D eICU-CRD Data and Preprocessing

In terms of clinical applications of AI, patient discharge status has been a popular problem to address, as it can directly influence clinical decision-making, resource allocation, and healthcare costs.

Here, the task was to predict the discharge status of a patient during the course of an ICU stay. Using similar inclusion and exclusion criteria to those used in a previous study [28], we selected adult patients (age > 18) with a minimum of 15 ICU records, and grouped these records into 1 hour windows. We removed any samples that did not have a clear discharge status (i.e., anything that was not "alive" or "expired").

Further preprocessing was performed to remove samples with any missing values, one-hot encode categorical features, and standardize all continuous features to have a mean of 0 and a standard deviation of 1.

We used a 60:20:20 training, validation, and test ratio, resulting in 49,305 training, 16,436 validation, and 16,436 test samples, respectively. As before, the training set was used for model development, hyperparameter selection, and training; the validation set is used for continous validation and threshold adjustment; and after successful development and training, the held-out test set was used to evaluate the performance of the final model.

Supplementary Table 5: Summary of number of patients, death cases, and hospital case distribution for training, validation, and held-out test set cohorts used in ethnicity debiasing task.

	Training	Validation	Test
***n*, patients**	49305	16436	16436
***n*, death**	4501	1486	1486
**Ethnicity:**			
Caucasian (%)	40285 (81.7)	13514 (82.2)	13510 (82.2)
African American (%)	5704 (11.6)	1832 (11.1)	1869 (11.4)
Hispanic (%)	2073 (4.2)	665 (4.0)	667 (4.1)
Asian (%)	938 (1.9)	319 (1.9)	282 (1.7)
Native American (%)	305 (0.6)	106 (0.6)	108 (0.7)

Supplementary Table 6: Clinical predictors considered for predicting patient discharge status and patient diagnosis.

Category	Features
Demographic features	Gender, age, height, weight
Measurements at hospital admission	Non-invasive systolic blood pressure, non-invasive diastolic blood pressure, non-invasive mean arterial pressure, heart rate, supporting oxygen used at admission, blood oxygen saturation, Glasgow coma score, diagnosis at admission
Measurements at ICU admission	Glucose

E Hyperparameter Values

Supplementary Table 7: Hyperparameter values used in COVID-19 status prediction (ethnicity debiasing) task.

	Hyperparameters:
RL	
	Gamma = 0.1
	Learning Rate = 0.0001
	Epsilon range = [0.01,1]
	Dropout = 0.3
	Hidden nodes = 150
	Optimizer = Adam
ADV	
	Hidden nodes (predictor) = 100
	Hidden nodes (adversary) = 10
	Dropout = 0.3
	Alpha = 10
	Learning Rate = 0.0001
NN	
	Dropout = 0.3
	Hidden nodes = 20
	Learning rate = 0.1
	Optimizer = Adam
XGB	
	Learning rate = 0.1
	N estimators = 100
	Depth = 3
NN (weighted)	
	Dropout = 0.3
	Hidden nodes = 20
	Learning rate = 0.1
	Optimizer = Adam
XGB (weighted)	
	Learning rate = 0.1
	N estimators = 100
	Depth = 3

Supplementary Table 8: Hyperparameter values used in patient ICU discharge status (ethnicity debiasing) task.

	Hyperparameters:
RL	
	Gamma = 0.1
	Learning Rate = 0.00009
	Epsilon range = [0.01,1]
	Dropout = 0.3
	Hidden nodes = 500
	Optimizer = Adam
ADV	
	Hidden nodes (predictor) = 200
	Hidden nodes (adversary) = 10
	Dropout = 0.3
	Alpha = 1
	Learning Rate = 0.0001
NN	
	Dropout = 0.3
	Hidden nodes = 200
	Learning rate = 0.1
	Optimizer = Adam
XGB	
	Learning rate = 0.1
	N estimators = 100
	Depth = 3
NN (weighted)	
	Dropout = 0.3
	Hidden nodes = 200
	Learning rate = 0.1
	Optimizer = Adam
XGB (weighted)	
	Learning rate = 0.1
	N estimators = 100
	Depth = 3

Supplementary Table 9: Hyperparameter values used in COVID-19 status prediction (hospital-site debiasing) task.

	Hyperparameters:
RL	
	Gamma = 0.1
	Learning Rate = 0.00009
	Epsilon range = [0.01,1]
	Dropout = 0.3
	Hidden nodes = 100
	Optimizer = Adam
ADV	
	Hidden nodes (predictor) = 100
	Hidden nodes (adversary) = 10
	Dropout = 0.3
	Alpha = 1
	Learning Rate = 0.0001
NN	
	Dropout = 0.3
	Hidden nodes = 10
	Learning rate = 0.1
	Optimizer = Adam
XGB	
	Learning rate = 0.1
	N estimators = 100
	Depth = 3
NN (weighted)	
	Dropout = 0.3
	Hidden nodes = 10
	Learning rate = 0.1
	Optimizer = Adam
XGB (weighted)	
	Learning rate = 0.1
	N estimators = 100
	Depth = 3

F Threshold Values

Supplementary Table 10: Adjusted Threshold Values Used for COVID-19 status prediction (ethnicity debiasing).

Model	Threshold	
	0.85	0.9
RL	0.50150	0.49049
ADV	0.08417	0.05010
NN	0.07708	0.05506
XGB	0.04204	0.01301
NN (weighted)	0.39139	0.34034
XGB (weighted))	0.08308	0.03203

Supplementary Table 11: Adjusted Threshold Values Used for patient ICU discharge status prediction (ethnicity debiasing).

Model	Threshold	
	0.85	0.9
RL	0.45445	0.44645
ADV	0.06814	0.04609
NN	0.05105	0.02803
XGB	0.06006	0.04304
NN (weighted)	0.37237	0.23023
XGB (weighted)	0.32332	0.24625

Supplementary Table 12: Adjusted Threshold Values Used for COVID-19 status prediction (hospital-site debiasing).

Model	Threshold	
	0.85	0.9
RL	0.47347	0.46747
ADV	0.05411	0.03206
NN	0.05405	0.03203
XGB	0.03407	0.01804
NN (weighted)	0.40040	0.31231
XGB (weighted)	0.13514	0.06306

G Additional Results

G.1 Previous Studies

Supplementary Table 13: Previously published COVID-19 status prediction results. using same datasets and patient cohorts. Sensitivity, specificity, and AUROC shown, alongside 95% confidence intervals, unless otherwise specified.

Test Set	Sensitivity	Specificity	AUROC
**Soltan et al., 2022.**			
Method: XGBoost + SMOTE + Threshold Adjustment (0.9)			
OUH	0.857 (SD 0.009)	0.686 (SD 0.022)	0.878 (SD 0.001)
PUH	0.841 (0.825-0.857)	0.713 (0.709 -0.718)	0.872 (0.863 -0.882)
UHB	0.788 (0.748-0.824)	0.747 (0.738 -0.755)	0.858 (0.838 -0.878)
BH	0.743 (0.666-0.807)	0.848 (0.825 0. 869)	0.881 (0.851- 0.912)
**Yang et al., 2022.**			
Method: Neural Network + SMOTE + Threshold Adjustment (0.85)			
OUH	0.844 (0.828-0.860)	0.710 (0.704-0.717)	0.777 (0.765-0.789)
PUH	0.857 (0.842-0.873)	0.672 (0.667-0.677)	0.765 (0.752-0.777)
UHB	0.847 (0.814-0.881)	0.716 (0.708-0.725)	0.782 (0.756-0.808)
BH	0.847 (0.789-0.906)	0.822 (0.799-0.845)	0.835 (0.793-0.876)
**Yang et al., 2022.**			
Method: Neural Network + Threshold Adjustment (0.85)			
OUH	0.762 (0.744-0.781)	0.844 (0.839-0.849)	0.878 (0.868-0.888)
PUH	0.633 (0.585-0.681)	0.903 (0.897-0.910)	0.861 (0.837-0.885)
UHB	0.714 (0.621-0.807)	0.854 (0.839-0.870)	0.878 (0.832-0.924)
BH	0.724 (0.561-0.887)	0.908 (0.869-0.948)	0.880 (0.798-0.963)

G.2 Debiasing Ethnicity

Supplementary Table 14: Equalized Odds evaluation for ethnicity bias and COVID-19 status prediction test results across different models and test sets, optimized to sensitivities of 0.85. Equalized odds results are reported as the SD of true positive and false positive rates across all ethnicity labels, with red- and blue-bolded values denoting the the best and second best scores, respectively. Classification metrics are reported alongside 95% confidence intervals (CIs), with bolded values denoting best scores achieved on each test set.

Test Set	Model	EO (TP)	EO (FP)	Sensitivity	Specificity	PPV	NPV	F1	AUROC
PUH									
	RL	**0.065**	0.049	0.819 (0.800-0.839)	0.640 (0.635-0.646)	0.109 (0.103-0.114)	0.985 (0.983-0.987)	0.192	0.834 (0.821-0.847)
	ADV	0.073	**0.024**	0.829 (0.810-0.849)	0.698 (0.692-0.703)	0.128 (0.121-0.135)	0.987 (0.986-0.989)	0.222	0.865 (0.853-0.877)
	NN	**0.062**	0.028	0.848 (0.830-0.867)	0.704 (0.699-0.710)	0.133 (0.126-0.140)	0.989 (0.987-0.990)	0.230	0.875 (0.863-0.886)
	XGB	0.207	0.028	0.835 (0.816-0.854)	0.740 (0.735-0.746)	0.147 (0.139-0.154)	0.988 (0.987-0.990)	0.250	0.882 (0.871-0.893)
	NN (weighted)	**0.062**	0.028	0.861 (0.843-0.878)	0.702 (0.696-0.707)	0.134 (0.127-0.141)	0.989 (0.988-0.991)	0.231	0.876 (0.864-0.887)
	XGB (weighted)	0.131	**0.026**	0.843 (0.824-0.862)	0.742 (0.737-0.747)	0.149 (0.141-0.156)	0.989 (0.987-0.990)	0.253	0.882 (0.871-0.894)
UHB									
	RL	**0.066**	0.039	0.844 (0.806-0.883)	0.712 (0.703-0.722)	0.108 (0.097-0.120)	0.991 (0.989-0.993)	0.192	0.849 (0.824-0.875)
	ADV	0.088	0.039	0.824 (0.784-0.864)	0.757 (0.747-0.766)	0.123 (0.110-0.136)	0.990 (0.988-0.993)	0.214	0.865 (0.840-0.889)
	NN	0.088	0.069	0.830 (0.790-0.869)	0.741 (0.732-0.750)	0.117 (0.104-0.130)	0.991 (0.988-0.993)	0.205	0.868 (0.843-0.892)
	XGB	0.096	**0.028**	0.761 (0.716-0.806)	0.795 (0.786-0.803)	0.133 (0.118-0.148)	0.988 (0.985-0.990)	0.226	0.854 (0.828-0.879)
	NN (weighted)	0.089	**0.036**	0.821 (0.781-0.862)	0.774 (0.765-0.783)	0.131 (0.117-0.145)	0.991 (0.988-0.993)	0.226	0.866 (0.841-0.890)
	XGB (weighted)	**0.056**	0.046	0.784 (0.741-0.827)	0.762 (0.753-0.771)	0.120 (0.107-0.134)	0.988 (0.986-0.991)	0.208	0.858 (0.833-0.883)
BH									
	RL	**<0.001**	**0.024**	0.877 (0.822-0.932)	0.817 (0.793-0.841)	0.399 (0.344-0.454)	0.979 (0.970-0.989)	0.549	0.923 (0.892-0.954)
	ADV	**0.051**	0.030	0.819 (0.755-0.883)	0.865 (0.844-0.886)	0.457 (0.395-0.520)	0.972 (0.961-0.983)	0.587	0.912 (0.879-0.945)
	NN	0.053	0.035	0.841 (0.780-0.902)	0.861 (0.840-0.883)	0.457 (0.395-0.518)	0.975 (0.965-0.985)	0.592	0.912 (0.879-0.945)
	XGB	0.088	**0.010**	0.848 (0.788-0.908)	0.845 (0.822-0.867)	0.432 (0.373-0.491)	0.976 (0.965-0.986)	0.572	0.908 (0.875-0.942)
	NN (weighted)	**0.051**	0.033	0.826 (0.763-0.889)	0.873 (0.852-0.894)	0.475 (0.412-0.538)	0.973 (0.962-0.984)	0.603	0.918 (0.886-0.950)
	XGB (weighted)	0.179	0.028	0.841 (0.780-0.902)	0.815 (0.791-0.839)	0.387 (0.332-0.442)	0.974 (0.963-0.984)	0.530	0.897 (0.861-0.932)

Supplementary Table 15: Equalized Odds evaluation for ethnicity bias and Patient ICU discharge prediction test results across different models, optimized to sensitivities of 0.85. Equalized odds results are reported as the SD of true positive and false positive rates across all ethnicity labels, with red- and blue-bolded values denoting the the best and second best scores, respectively. Classification metrics are reported alongside 95% confidence intervals (CIs), with bolded values denoting best scores achieved on the test set.

Model	EO (TP)	EO (FP)	Sensitivity	Specificity	PPV	NPV	F1	AUROC
RL	**0.042**	**0.017**	0.845 (0.827-0.863)	0.645 (0.637-0.653)	0.201 (0.191-0.211)	0.975 (0.972-0.978)	0.325	0.829 (0.816-0.841)
ADV	0.056	**0.020**	0.833 (0.815-0.852)	0.720 (0.713-0.727)	0.239 (0.228-0.250)	0.976 (0.973-0.979)	0.372	0.861 (0.849-0.873)
NN	**0.042**	0.024	0.843 (0.825-0.861)	0.688 (0.681-0.695)	0.222 (0.212-0.233)	0.977 (0.974-0.979)	0.352	0.847 (0.835-0.859)
XGB	0.099	0.035	0.831 (0.813-0.850)	0.748 (0.741-0.755)	0.258 (0.246-0.270)	0.977 (0.974-0.979)	0.394	0.875 (0.863-0.886)
NN (weighted)	**0.048**	0.026	0.834 (0.815-0.852)	0.700 (0.693-0.708)	0.227 (0.216-0.238)	0.976 (0.973-0.978)	0.357	0.843 (0.831-0.856)
XGB (weighted)	0.075	0.026	0.829 (0.811-0.848)	0.738 (0.730-0.745)	0.250 (0.239-0.262)	0.976 (0.973-0.979)	0.385	0.871 (0.860-0.883)

G.3 Debiasing Hospital

Supplementary Table 16: Equalized Odds evaluation for hospital bias and COVID-19 status prediction test results across different models and test sets, optimized to sensitivities of 0.85. Equalized odds results are reported as the SD of true positive and false positive rates across all hospital labels, with red- and blue-bolded values denoting the the best and second best scores, respectively. Classification metrics are reported alongside 95% confidence intervals (CIs), with bolded values denoting best scores achieved on the test set.

Model	EO (TP)	EO (FP)	Sensitivity	Specificity	PPV	NPV	F1	AUROC
RL	**0.014**	**0.033**	0.835 (0.813-0.858)	0.754 (0.746-0.761)	0.209 (0.197-0.222)	0.983 (0.981-0.986)	0.335	0.879 (0.865-0.892)
ADV	0.022	0.037	0.835 (0.813-0.858)	0.758 (0.751-0.765)	0.212 (0.200-0.225)	0.983 (0.981-0.986)	0.339	0.882 (0.869-0.896)
NN	**0.020**	0.042	0.830 (0.807-0.852)	0.796 (0.789-0.803)	0.241 (0.227-0.255)	0.984 (0.981-0.986)	0.373	0.891 (0.879-0.904)
XGB	0.026	0.039	0.830 (0.808-0.853)	0.817 (0.811-0.824)	0.262 (0.247-0.277)	0.984 (0.982-0.986)	0.398	0.900 (0.887-0.912)
NN (weighted)	0.036	**0.035**	0.833 (0.811-0.856)	0.789 (0.782-0.796)	0.236 (0.222-0.249)	0.984 (0.981-0.986)	0.368	0.894 (0.881-0.906)
XGB (weighted)	0.033	0.036	0.830 (0.807-0.852)	0.805 (0.798-0.811)	0.249 (0.235-0.263)	0.984 (0.981-0.986)	0.383	0.901 (0.888-0.913)
