# Supplementary material for: Algorithmic fairness and bias mitigation for clinical machine learning with deep reinforcement learning
Source: Nat Mach Intell. 2023 Jul 31;5(8):884–94. doi: 10.1038/s42256-023-00697-3 (PMC10442224; doi:10.1038/s42256-023-00697-3)
Supplement: Supplementary file 2 — Reporting Summary [file 42256_2023_697_MOESM2_ESM.pdf]

nature research

Corresponding author(s): Jenny Yang

Last updated by author(s): Jun 6, 2023

Reporting Summary

Nature Research wishes to improve the reproducibility of the work that we publish. This form provides structure for consistency and transparency in reporting. For further information on Nature Research policies, see our Editorial Policies and the Editorial Policy Checklist.

Statistics

For all statistical analyses, confirm that the following items are present in the figure legend, table legend, main text, or Methods section.

n/a	Confirmed	
☐	☒	The exact sample size (*n*) for each experimental group/condition, given as a discrete number and unit of measurement
☐	☒	A statement on whether measurements were taken from distinct samples or whether the same sample was measured repeatedly
☐	☒	The statistical test(s) used AND whether they are one- or two-sided<br>*Only common tests should be described solely by name; describe more complex techniques in the Methods section.*
☒	☐	A description of all covariates tested
☐	☒	A description of any assumptions or corrections, such as tests of normality and adjustment for multiple comparisons
☐	☒	A full description of the statistical parameters including central tendency (e.g. means) or other basic estimates (e.g. regression coefficient) AND variation (e.g. standard deviation) or associated estimates of uncertainty (e.g. confidence intervals)
☒	☐	For null hypothesis testing, the test statistic (e.g. *F*, *t*, *r*) with confidence intervals, effect sizes, degrees of freedom and *P* value noted<br>*Give P values as exact values whenever suitable.*
☒	☐	For Bayesian analysis, information on the choice of priors and Markov chain Monte Carlo settings
☐	☒	For hierarchical and complex designs, identification of the appropriate level for tests and full reporting of outcomes
☒	☐	Estimates of effect sizes (e.g. Cohen's *d*, Pearson's *r*), indicating how they were calculated

Our web collection on statistics for biologists contains articles on many of the points above.

Software and code

Policy information about availability of computer code

Data collection	No software was used here.
Data analysis	Models were implemented using Python (v3.6.9). Scikit Learn (v0.24.1) was used for standardization, median imputation, and calculating performance metrics. Performance metrics were calculated using Scikit Learn and manually programmed. XGBoost baseline models were implemented using the XGBoost library (v1.3.3). Neural network baseline models were implemented using Keras (v2.6.0). Reinforcement learning was set up using Tensorflow (v2.6.2). All models were run using an Intel Xeon E-2146G Processor (CPU: 6 cores, 4.50 GHz max frequency). Code can be found in: https://github.com/yangjenny/reinforcement_learning_bias_mitigation and https://codeocean.com/capsule/6724009

For manuscripts utilizing custom algorithms or software that are central to the research but not yet described in published literature, software must be made available to editors and reviewers. We strongly encourage code deposition in a community repository (e.g. GitHub). See the Nature Research guidelines for submitting code & software for further information.

Data

Policy information about availability of data

All manuscripts must include a data availability statement. This statement should provide the following information, where applicable:

- Accession codes, unique identifiers, or web links for publicly available datasets
- A list of figures that have associated raw data
- A description of any restrictions on data availability

Data from OUH studied here are available from the Infections in Oxfordshire Research Database (https://oxfordbrc.nihr.ac.uk/research-themes/modernising-medical-microbiology-and-big-infection-diagnostics/infections-in-oxfordshire-research-database-iord/), subject to an application meeting the ethical and

governance requirements of the Database. Data from UHB, PUH and BH are available on reasonable request to the respective trusts, subject to HRA requirements. The eICU Collaborative Research Database is available online at https://www.physionet.org/content/eicu-crd/2.0/

Field-specific reporting

Please select the one below that is the best fit for your research. If you are not sure, read the appropriate sections before making your selection.

☒ Life sciences ☐ Behavioural & social sciences ☐ Ecological, evolutionary & environmental sciences

For a reference copy of the document with all sections, see nature.com/documents/nr-reporting-summary-flat.pdf

Life sciences study design

All studies must disclose on these points even when the disclosure is negative.

Sample size	We obtained all presentations/admissions to emergency rooms across four United Kingdom National Health Service Trusts. Sample sizes used are the real/complete set of patient presentations during the period of data extraction.
Data exclusions	We excluded patients who opted out of electronic health record (EHR) research and those who did not receive laboratory blood tests or were younger than 18 years of age. This criteria was pre-established, and also used in previous studies which used this data.
Replication	We demonstrated and discussed experimental and conceptual reproducibility through performing external validation across three independent hospital trusts. We also show model generalization on an additional independent domain. All attempts at replication were successful.
Randomization	Groups were based on NHS Trust. Machine learning training, validation and testing groups were randomly assigned.
Blinding	Blinding is not applicable, as this is now a randomized or clinical trial. The data uses PCR testing as the gold standard for classification of COVID-19; thus, there is no risk of any participant bias.

Reporting for specific materials, systems and methods

We require information from authors about some types of materials, experimental systems and methods used in many studies. Here, indicate whether each material, system or method listed is relevant to your study. If you are not sure if a list item applies to your research, read the appropriate section before selecting a response.

Materials & experimental systems

n/a	Involved in the study	
☒	☐	Antibodies
☒	☐	Eukaryotic cell lines
☒	☐	Palaeontology and archaeology
☒	☐	Animals and other organisms
☐	☒	Human research participants
☒	☐	Clinical data
☒	☐	Dual use research of concern

Methods

n/a	Involved in the study	
☒	☐	ChIP-seq
☒	☐	Flow cytometry
☒	☐	MRI-based neuroimaging

Human research participants

Policy information about studies involving human research participants

Population characteristics	The training cohort had a 11.9% COVID-19 prevalence. 49366 patients were included across 3 validation cohorts, of whom 2588 had a positive confirmatory test for SARS-CoV-2. Patients admitted to PUH and BH trusts had similar ages (69 years, IQR 34, for PUH and 68 years, 34, for BH), whereas patients admitted to UHB were younger (63 years, 37). A higher proportion of patients admitted to UHB were women (5462 [53·1%] of 10293) than those admitted to PUH (17054 [45·0%] of 37896) and BH (549 [46·7%] of 1177) and reported being of South Asian ethnicity (1357 [13·2%] UHB). vs 170 [0·5%] PUH and 71 (6·0%) BH;). COVID-19 prevalence was highest in the BH cohort owing to the evaluation timeline spanning the second UK pandemic wave (11·1% vs 5·3% in PUH and 4·3% in UHB).
Recruitment	This data was already available in a pre-established database. All patient presentations were included unless they did not meet the inclusion criteria.
Ethics oversight	United Kingdom National Health Service (NHS) approval via the national oversight/regulatory body, the Health Research Authority (HRA), has been granted for development and validation of artificial intelligence models to detect Covid-19 (CURIAL; NHS HRA IRAS ID: 281832).

Note that full information on the approval of the study protocol must also be provided in the manuscript.
